# Three-dimensional position changes of unopposed molars before implant rehabilitation: a short-term retrospective cohort analysis

**DOI:** 10.1186/s12903-022-02619-y

**Published:** 2022-12-03

**Authors:** Anqi Wang, Jie Cao, Haoyun Zhang, Bo Zhang, Gang Yang, Wenjie Hu, Kwok‑Hung Chung

**Affiliations:** 1 Department of Periodontology, Peking University School and Hospital of Stomatology, National Engineering Laboratory for Digital and Material Technology of Stomatology, Beijing Key Laboratory of Digital Stomatology, National Clinical Research Center for Oral Disease, 22 Zhongguancun Avenue South, Haidian District, Beijing, 100081 People’s Republic of China; 2 Present Address: Department of Periodontology, Qingdao Stomatology Hospital Affiliated to Qingdao University, No.17 Dexian Road, Shinan District, Shandong Province, Qingdao, 266001 People’s Republic of China; 3 Department of Stomatology, Beijing Friendship Hospital, Capital Medical University, Beijing, People’s Republic of China; 4 Department of Restorative Dentistry, University of Washington, Seattle, WA USA

**Keywords:** Overeruption, Tipping, Periodontitis, Edentulous, Radiology

## Abstract

**Objective:**

To investigate the spatial changes of unopposed molars within the period between the antagonist extraction and the final implant restoration using data from cone beam computed tomography.

**Methods:**

A total of 59 patients with 68 unopposed molars were included in this study. Three-dimensional models reconstructed from cone beam computed tomography data before and after loss of the antagonist were superimposed to measure the spatial changes. The overeruption and tipping of target teeth were calculated by coordinate values.

**Results:**

The result of overeruption over the study period (9.2 ± 4.3 months) was expressed by two values: the mean overeruption of molar cups (0.432 mm) and the maximum overeruption of cusps (0.753 mm), which were statistically significant compared to the baseline level (*p* < 0.001). The average tipping was 1.717 degrees in the buccal direction.

**Conclusions:**

Unopposed molars displayed overeruption throughout the study period (9.2 ± 4.3 months), which indicates that the clinicians should pay attention to the possibilities of overeruption and make appropriate interventions in their clinical practice. The establishment of three-dimensional measuring methods using cone beam computed tomography data helps analyze spatial changes.

## Background

Periodontal disease is one of the two major causes of tooth loss. Molars are commonly involved because of the burden of occlusal function, difficulties in plaque control, and complex anatomical structures such as root furcation [1]. Implant therapy is one of the most common treatment options to replace missing teeth. Severe periodontitis often causes significant hard and soft tissue defects, therefore usually requiring soft and hard tissue augmentation or ridge preservation to maintain the alveolar socket contour before implant placement [2]. Nevertheless, there must be a healing period of at least six months before implant placement surgery, and the entire implant restoration period is about 9–12 months [3, 4].

Missing molars that remain unrestored can break the balance within the stomatognathic system; with this in mind, there is a common belief among dentists that spatial changes can occur in the molars adjacent or opposed to the edentulous areas [5, 6]. However, some relevant studies have been skeptical of the amount of occlusal change expected after tooth extraction, and whether such changes have a significant effect on long-term occlusal stability [7, 8]. The overeruption of unopposed molars may cause occlusal interferences and complicate the replacement of the missing tooth [9–11]. This will be a critical consideration when implant therapy is planned [12]. Previous studies reported the extent and prevalence of overeruption in unopposed molars [11, 13–16]. However, limited by their cross-sectional designs, it is hard to distinguish whether overeruption is a true positional change or a pre-existing difference in tooth position. A few cohort studies focused on spatial changes in the vertical direction of molars before and after antagonist loss [17–19]. An average overeruption of 0.8–0.9 mm was found after a ten-year follow-up. Several factors were reported associated with the spatial changes of overeruption of tooth, such as the age when the antagonist teeth were lost, arch location, and severity/duration of periodontal disease [7, 8, 11, 14, 18, 20]. Nevertheless, the overeruption of a molar is potentially a complicated spatial movement, involving not only vertical migration, but also tipping and rotation [21]. There is a lack of research on the overeruption of unopposed natural teeth within the implant restoration therapy period. Cone-beam computed tomography (CBCT) is now generally used to obtain 3-dimensional (3D) information for related measurements [22]. Therefore, 3D measurement methods using CBCT data will be utilized to monitor the spatial changes of a molar comprehensively and directly.

The aim the study was to establish a method for 3D measurements of overeruption based on CBCT measurements to investigate the spatial changes of unopposed molars within the period between the antagonist extraction and the final implant restoration.

## Methods

### Subjects

The study is a retrospective cohort study. Subjects were selected from 321 patients with one or more missing molars seeking replacement and rehabilitation by implant restoration; these patients visited the Department of Periodontology, Peking University School and Hospital of Stomatology from January 2013 to December 2020. Molars opposing the missing molars were selected as target teeth. The inclusion criteria were as follows: (a) ≥ 25 years old; (b) patients with at least one molar missing, with records showing the time of extraction clearly, as well as evidence of the existing antagonist teeth; (c) the unopposed teeth had no contact with any opposite teeth according to the plaster models and clinical occlusal photographs; (d) there were CBCT radiographs including the target teeth within three months before extraction of the antagonist (baseline) and after extraction. The patients were excluded if they (a) wore an occlusal guard; (b) had a history of orthodontic treatment prior to the tooth extraction; (c) had history of drug therapy or radiation therapy that severely affects bone metabolism; (d) had uncontrolled systemic diseases. All the patients in the study had periodontitis. 69.5% of the patients were diagnosed as stage 3, grade C according to new classification [23]. Initially, 146 patients were not included due to lacking records showing the time of extraction. Then, 6 patients were excluded because of orthodontic history, or the tooth extracted without any antagonist teeth. The screening for CBCT radiographs meeting the requirements further reduced the sample size by 67 patients. Then, plaster models and clinical occlusal photographs were used to identify whether the unopposed teeth had no contact with any opposite teeth; 43 patients were excluded at this step. Finally, a total of 59 patients with 68 target teeth were included in this study. Written informed consent about the application of medical information for research was obtained from all participants. The study was conducted in full accordance with the ethical principles established in the World Medical Association Declaration of Helsinki of 1975 as revised in 2000 and approved by the Institutional Review Boards of the (Approval Number: 201949134).

### 3D reconstruction of CBCT and coordinates establishment for measurements

**Fig. 1:**
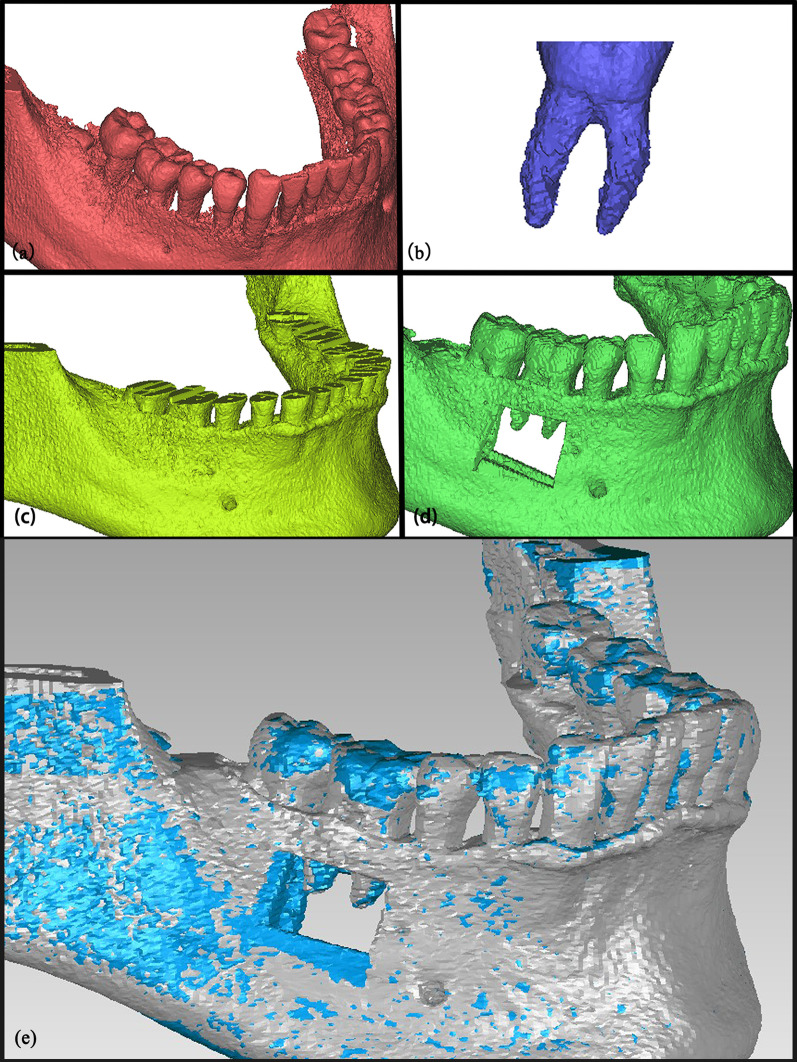
Processing in extracting CBCT 3D model information. **a** Crown: extracting 3D model under the experience threshold of crown; **b** Root: extracting 3D model under experience threshold of root with removing part of cusps; **c** Alveolar bone: extracting 3D model under the experience threshold of alveolar bone with removing the crown part; **d** Model for measurement after uniting; **e** The alignment of preoperative and postoperative CBCT images

All participants received a pre-operative CBCT examination using a New Tom 9000 CBCT (Aperio Services, Italy) within 3 months before extracting the antagonist of the target tooth to confirm the indication for extraction or the possibilities of socket preservation. All scans were taken at 110 kV, 12–17 mA, with 0.1–0.3 mm slice thickness and pixel size of 0.125 mm, and this pre-operative data was recorded as T_o_. The postoperative CBCT was performed before the definitive restoration of the edentulous space using the same CBCT machine and protocol; this time was documented as T_n_. The Digital Imaging and Communications in Medicine (DICOM) file from the CBCT examination was imported and merged in a volumetric imaging software (Mimics 20.0, Materialise, Belgium), in which 3D images of models were constructed. Thresholding was used to obtain the 3D models under the applicable thresholds of crown, alveolar bone, and root. The mask in each threshold was named as "Crown", "Root", and "Alveolar bone", respectively (Fig. 1a–c). The unit in Boolean operations was used to unite the masks, and a window around the apical region of target tooth was made to expose the root in the model (Fig. 1d). The final 3D models were generated into the Standard Tessellation Language (STL) files. Postoperative images (T_n_) were superimposed with pre‐operative (T_o_) CBCT images using automated surface best fit aligning with the iterative closest point algorithm in the treatment evaluation mode of a reverse engineering software (Geomagic Control 2015, 3D Systems, Inc., USA) (Fig. 1e). All crowns of the dentition, except the target teeth and the stable alveolar bone area not containing the crest of the alveolar ridge and the open window area, were selected as the align area.

**Fig. 2:**
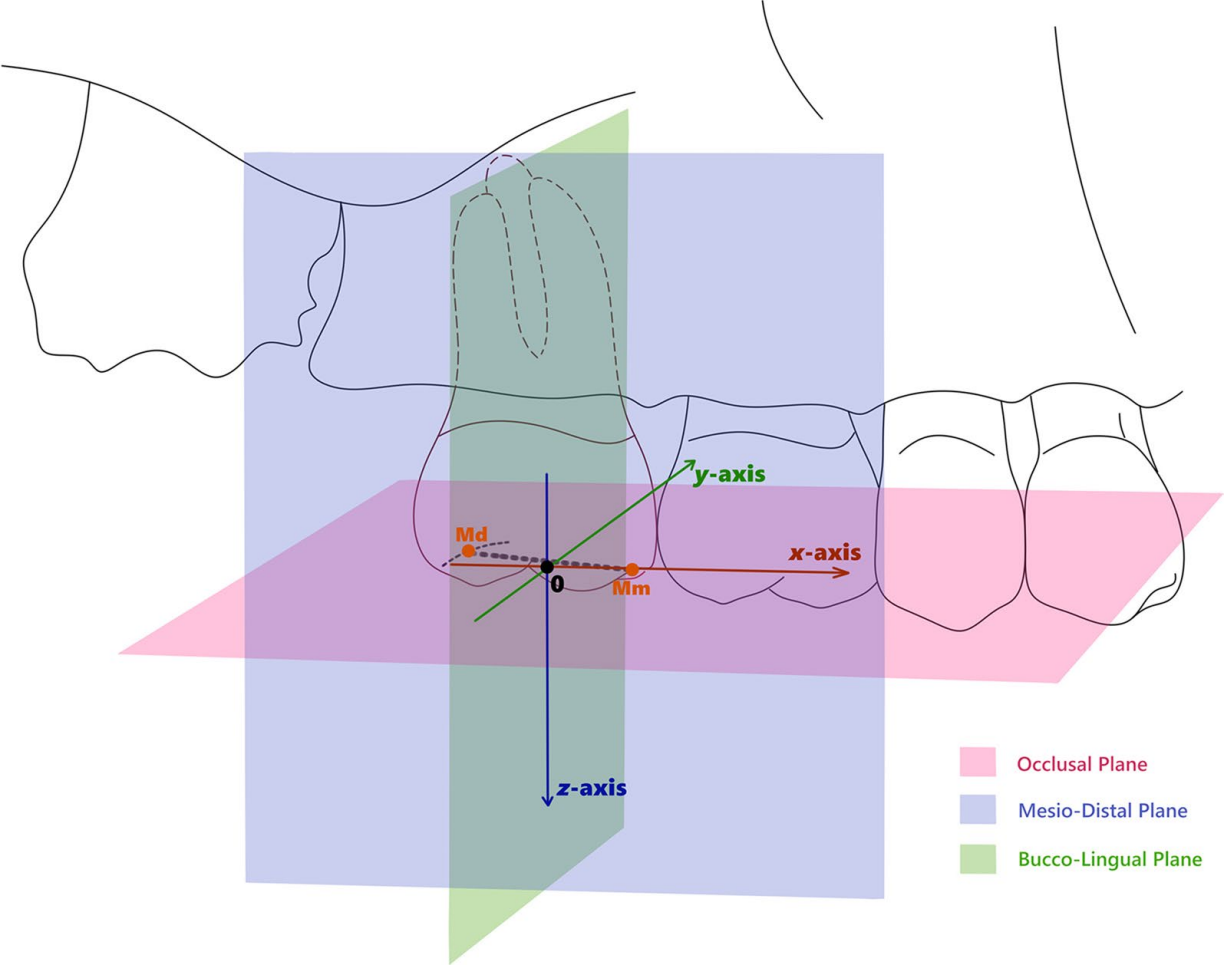
Determination of three reference planes and establishment of 3D coordinate system of the target tooth. 0, original point; Mm, midpoint of mesial marginal crest; Md, midpoint of distal marginal crest

According to the method established by Chen et al. [24], the 3D coordinates of target teeth were established by confirming three mutually perpendicular reference planes on the baseline model (T_o_): Occlusal Plane, Mesio-Distal Plane, and Bucco-Lingual Plane (Fig. 2). Occlusal Plane was calculated by matching all the cusp tips of the posterior teeth in the quadrant of target tooth. A line perpendicular to the Occlusal Plane and across the midpoint of the line connecting the midpoint of the mesial and distal marginal crests was defined as the z-axis. The original point was the meeting point of the z-axis and Occlusal Plane. The original point was assigned as 0. The Mesio-Distal Plane was a plane passing through the z-axis and the midpoint of the distal marginal crest. The Bucco-Lingual Plane was a plane passing through the original point and was perpendicular to the two aforementioned planes. The positive direction of *x*-axis, *y*-axis, and *z*-axis was set in mesial, lingual, and occlusal direction, respectively.

### Determination of the reference points and parameters

**Fig. 3:**
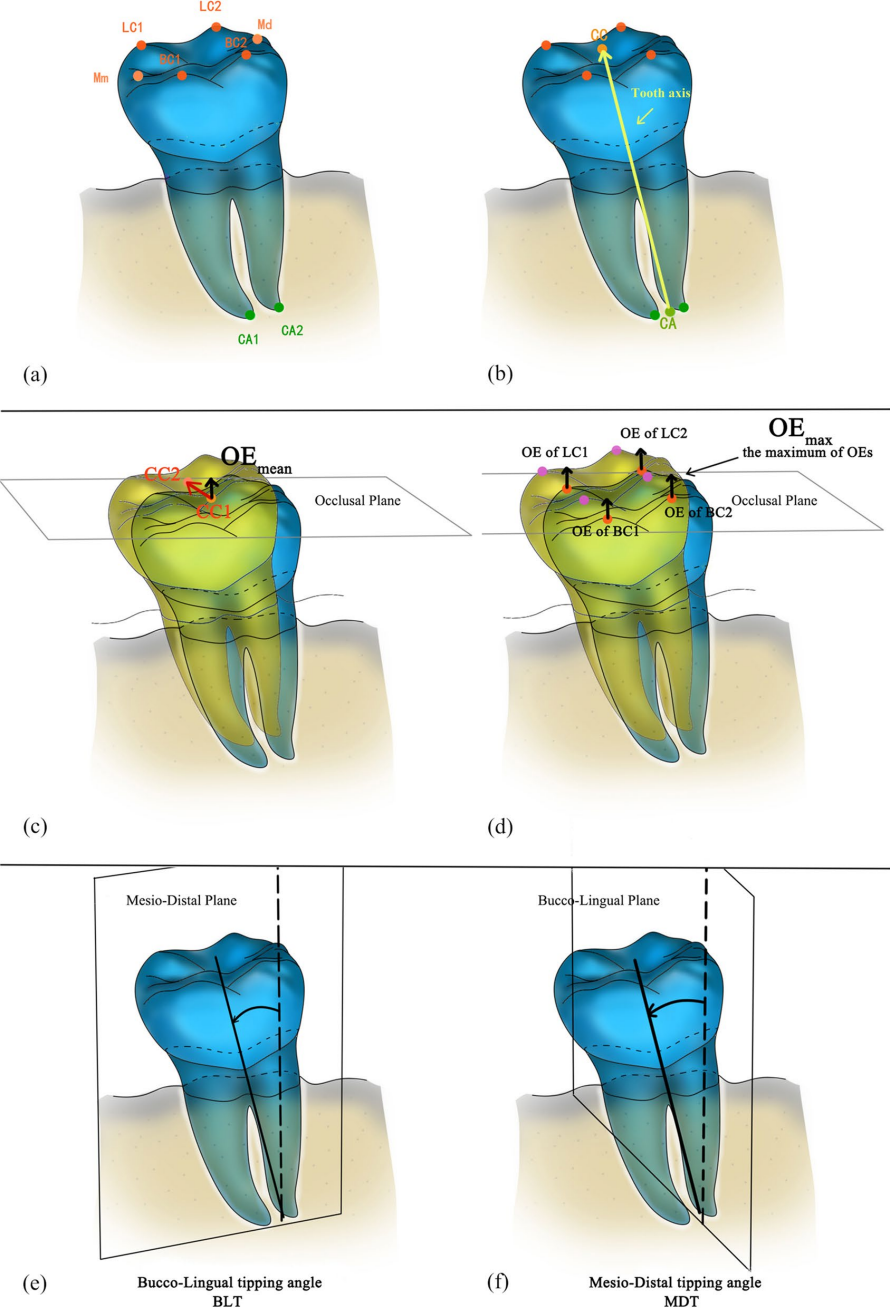
Determination of reference points and parameters. **a** LC1, LC2, BC1, and BC2 were cusp tips, in which LC and BC means lingual cusp and buccal cusp; Mm and Md were midpoints of the mesial and distal marginal crests, respectively. **b** CC: centroid of cusp tips; CA: centroid of root apexes; Tooth axis, line from CA to CC. **c** OE_mean,_ mean overeruption of cusps **d** OE_max,_ maximum overeruption of cusps. **e** BLT, Bucco-Lingual tipping angle. **f** MDT, Mesio-Distal tipping angle

Points of reference were determined on each 3D model (Fig. 3a). Cusp tips were used as reference points, and the average coordinate values of all the cusp tips were taken to obtain the centroid of cusp tips (CC). The average coordinate values of the root apex were taken to obtain the centroid of apex (CA). The midpoints of the mesial and distal marginal crest were assigned as Mm and Md, respectively. The vector from CA to CC was defined as the tooth axis (Fig. 3b). The parameters related to spatial changes such as overeruption and tipping were shown from Fig. 3c to f. The values of the parameters were derived from coordinate calculations. The detailed definition of each parameter is as follows:

As the vector CC1 to CC2 reflects the displacement of the centroid of cusp tips (Fig. 3c), its value in the occlusal direction can be used to describe the overeruption. Mean overeruption of cusps (OE_mean_): the occlusal movements of the centroid of cusp tips in the z-axis direction between T_n_ and T_o_ (Fig. 3c). Maximum overeruption of cusps (OE_max_): the maximum displacements among cusp tips in the z-axis direction (Fig. 3d). Bucco-Lingual tipping angle (BLT) represents the angle between the tooth axis and Mesio-Distal Plane (Fig. 3e). Mesio-Distal tipping angle (MDT) represents the angle between the tooth axis and Bucco-Lingual Plane (Fig. 3f). The difference between the angle of tipping at T_n_ and T_o_ is the outcome index, named as Bucco-Lingual tipping change (cBLT) or Mesio-Distal tipping change (cMDT).

### Statistical analysis

Statistical evaluation was undertaken with the Statistical Package for Social Sciences, version 22.0 (SPSS Inc., Chicago, Illinois, USA). Statistical analyses were performed at tooth level. Data obtained from coordinate values were expressed in terms of means and ranges. Frequency (percentages) were computed for categorical variables. A one-sample *t-*test was performed, and the zero value was used as a reference to compare with the averages of the evaluated parameters. As the normal distribution of the parameters was observed using the graphical method, one-way analysis of variance (ANOVA) was used to compare the degree of overeruption and tipping of different molar types based on a significant level at α = 0.05. The specific data distribution was displayed by bar chart, box chart, and line chart.

### Error of method

All assessments were repeated on 10 randomly selected subjects after 2 weeks. The consistency between the two measurements was assessed using intra-class correlation efficient (ICC), and a two-way mixed model was chosen. A high consistency of OE_mean_ and OE_max_ obtained from the two measurements (ICC = 0.965 and 0.973, respectively) was found. The size of the combined method error in locating the landmarks and the measuring procedure was evaluated using Dahlberg’s (1940) formula and the results were as follows: the OE_mean_ was 0.074 mm, and the OE_max_ was 0.092 mm, respectively. There was compliance with the STROBE checklist.

## Results

### Sample characteristics

A total of 59 patients with 68 teeth were included. Of these, 48 teeth belonged to male patients, and 20 teeth belonged to female patients. The mean age of the patients when the tooth was unopposed was 48.8 years (SD 8.5, range 30–78 years). The mean t_n_ was 9.1 months (SD 4.3). The results are shown in Table 1.

**Table 1:** Sample characteristics

Characteristics at tooth level	Mean ± standard deviation or frequency	Percentage (%)
*Sex*		
Male	48	70.60
Female	20	29.40
Age^†^ (year)	48.8 ± 8.5	
t_n_^‡^ (month)	9.1 ± 4.3	
*Tooth position*		
Maxilla	47	69.10
First molar	14	20.60
Second molar	33	48.50
Mandible	21	30.90
First molar	7	10.30
Second molar	14	20.60

A total of 59 patients with 68 teeth were included

^**†**^Age of patients at time of extraction

^**‡**^Length of time of target tooth remained unopposed

### Overeruption and tipping for all the target teeth

**Fig. 4:**
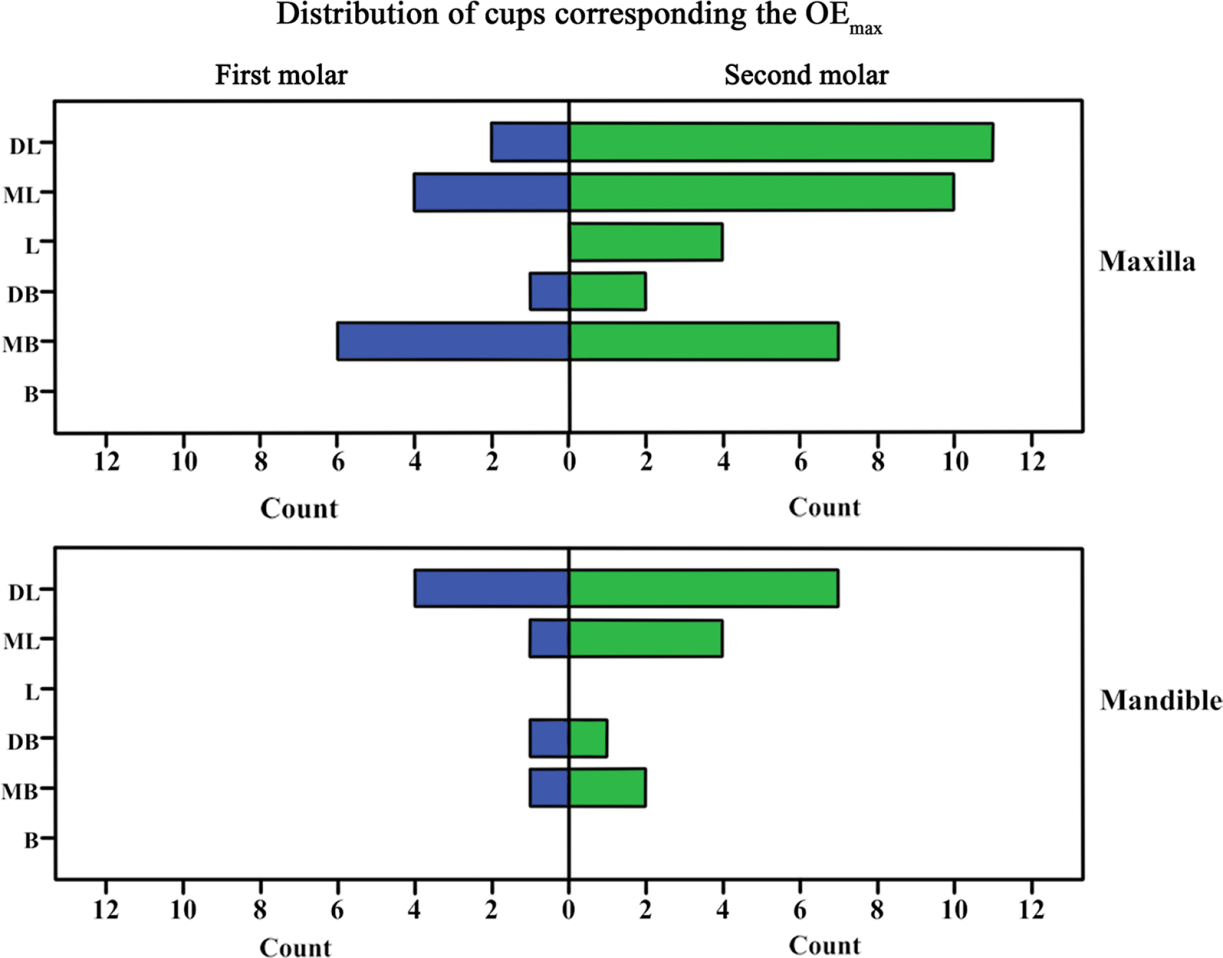
Distribution of cusps corresponding to the OE_max_. DL, distal-lingual cusp; ML, mesio-lingual cusp; DB, distal-buccal cusp; MB, mesio-buccal cusp; B, buccal cusp; MB, mesial-buccal cusp; L, lingual cusp

The average OE_mean_ of unopposed molars was 0.432 mm and OE_max_ was 0.753 mm, which were statistically significant (*p* < *0.001*). The average tipping was 1.717° in the buccal direction, and the cMDT (*p* = *0.103*) was not statistically significant (Table 2). The OE_max_ was mostly observed at lingual or palatal cusps (Fig. 4).

**Table 2:** Overeruption and tipping in unopposed molars

Mean ± standard deviation		Range		One sample *t*-test	
		Min	Max	*t*	*p*-values
OE_mean_ (mm)	0.432 ± 0.432	− 0.410	1.920	8.252	< 0.001*
OE_max_ (mm)	0.753 ± 0.523	0.034	2.555	11.891	< 0.001*
cMDT (°)	− 0.487 ± 2.180	− 6.247	4.053	− 1.660	0.103
cBLT (°)	− 1.717 ± 3.802	− 11.705	9.207	− 3.350	0.001*

The average tipping was in the buccal direction, which was statistically significant

*p < 0.05

*OE*_*mean*_ overeruption of cusps; *OE*_*max*_ maximum overeruption of cusps; *cMDT* Mesio-Distal tipping change; *cBLT* Bucco-Lingual tipping change

### Distribution of overeruption and tipping in different sites

**Fig. 5:**
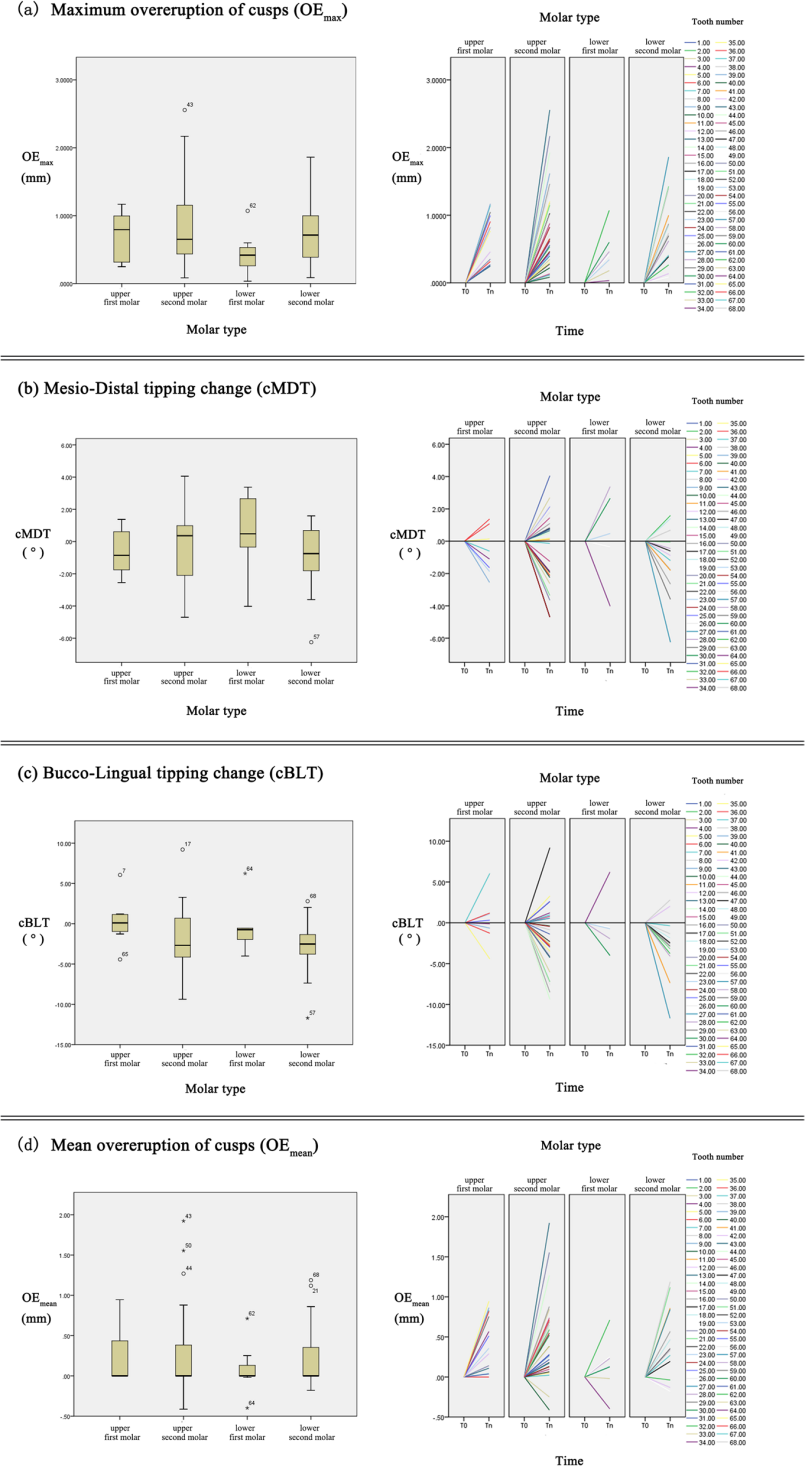
Distribution of overeruption and tipping in different sites of molars. **a**, **b**, **c**, and **d** were box diagrams and line charts that show distribution of overeruption and tipping in different sites

The distribution of overeruption and tipping in different sites is shown in Fig. 5. Most of the teeth (69.1%) showed buccal tipping, but the tipping directions were evenly distributed on both mesial and distal sides. One-way ANOVA revealed there were no significant differences for different molar types in OE_max_, cMDT, and cBLT. There were two upper second molars that had an OE_max_ of more than 2 mm, and the largest OE_max_ value (2.555 mm) appeared in only 9 months (Fig. 5b). Most of the teeth (72.1%) had an OE_max_ of less than 1 mm. The degrees of tipping varied with 90.0% of the distribution within 5 degrees. Teeth No. 64, No. 40 and No. 33 with negative values for OE_mean_ both corresponded to a large absolute value of cBLT (Fig. 5d and c).

## Discussion

IN the current study, the movement of the centroid of the cusp tips was measured in three dimensions by reconstructing and analyzing CBCT data. The results showed that molars without an antagonist underwent spatial changes or overeruption during the follow-up time (9.2 ± 4.3 months). This suggests that even if the molar was restored with implant-retained or conventional fixed or removable prosthesis within one year after extraction, its opposing tooth may have still had a small amount of overeruption towards the occlusal direction at the edentulous site. It is critical that when implant restorations are planned for molars that need to be extracted, the space management is considered.

CBCT is commonly used in clinical practice to obtain 3D information of oral hard tissue. The application and development of volume imaging software and reverse engineering software also makes CBCT data available for 3D measurements. Compared with 3D measurements based on digital model scanning, it is easier to obtain oral hard tissue information with CBCT images, including alveolar bone and root; CBCT images can also display the complete teeth movement instead of only focusing on the information of the crowns. When using CBCT imaging to obtain 3D digital model for measurements, the accuracy and quality of model should be considered. Selection of threshold is the key to distinguish the boundaries of soft and hard tissues, and between different hard tissues. The research on the accuracy of threshold segmentation indicates that multi-threshold segmentation could lead to smaller error compared with single threshold segmentation [25]. In this study, the different structures such as crown, root, alveolar bone were extracted in their experience thresholds and then united to form the final model; this ensures the clarity and accuracy of each part at the same time. The results of error analysis support the accuracy of this method in this 3D measurement study.

It is important for clinical research to determine the reference points and parameters to evaluate the overeruption. In the present investigation, the amount of overeruption was reflected by two parameters: mean overeruption of cusps and maximum overeruption of cusps. While the latter can reflect the maximum amount of tooth movement in the occlusal direction compared with its original position, the distribution of the maximum overeruption of cusps can reflect the tipping trend of overerupted teeth. Results of this study indicated that the mean overeruption of cusps was 0.432 mm and maximum overeruption of cusps was 0.753 mm during the study period (9.2 ± 4.3 months). Previous cohort studies observed the overeruption most in association with a long period of antagonist loss (6.9–12 years) and showed lager amount of overeruption (mean value was 0.8 to 0.9 mm) compared to this study [17–19]. The cohort study of Shugars et al. and Lindskog-Stokland et al. obtained tooth overgrowth information by two-dimensional imaging measurements [17, 19]. Because the measured mark points must be the most occlusal points on the cusps which are not overlapped in the dental films and panoramic radiographs, the overgrowth value is similar to the maximum overeruption of cusps in this investigation. The results of Lindskog-Stokland et al*.* showed approximately 0.9 mm of overeruption during the 12-year observation period for the unopposed molars, which was just a little bit lager than the results of this study [19]. It can be speculated that the extent of overeruption may be greatest in the initial period of time.

The results of the current study revealed that the average tipping was 1.7 degrees in the buccal direction, and the OE_max_ was mostly (69.1%) observed at lingual (palatal) cusps. This result is contrary to a previous study, whose results indicated greater eruption of the vestibular side of the molar and a simultaneous rotation of the molar in the transverse plane to the palatal direction [18]. The reason for this difference is not clear currently, but it may be related to the different study populations. The tipping movement of the unopposed molars may partially explain the negative value of the OE_mean_.

Participants in this study were patients with periodontitis. Periodontal conditions are associated with overeruption [9, 13]. With this in mind, a larger extent of overeruption was found in patients with periodontitis, although the sample size of patients with periodontitis included in these two studies was only 10 and 4 cases. Therefore, further investigation is required to analyze the relationship between local periodontal parameters and overeruption.

This study mainly focused on the description of overeruption and tipping value and has a small sample size. This limitation precludes a multivariate analysis with predictive factors, as well as including the patient’s age in the regression model. Predictive factors should be analyzed in further studies with larger sample sizes and targeted designs.

## Conclusions

1. A method of 3D measurements of overeruption based on CBCT imaging was established.
2. The overeruption of unopposed molars is a complicated spatial movement, involving not only vertical migration, but also tipping and rotation.
3. Teeth displayed overeruption over a short study period (9.2 ± 4.3 month).
4. Buccal displacement of unopposed molars was observed.

## Data Availability

The data sets used and/or analyzed during the current study are available from the corresponding author on reasonable request.

## Abbreviations

CBCT: Cone-beam computed tomography
3D: 3-Dimensional
DICOM: Digital imaging and communications in medicine
STL: Standard tessellation language
OE_mean_: Mean overeruption of cusps
OE_max_: Maximum overeruption of cusps
BLT: Bucco-lingual tipping angle
MDT: Mesio-Distal tipping angle
cBLT: Bucco-lingual tipping change
cMDT: Mesio-distal tipping change
ANOVA: Analysis of variance
ICC: Intra-class correlation efficient
